# Study of the Allocation of Regional Flood Drainage Rights in Watershed Based on Entropy Weight TOPSIS Model: A Case Study of the Jiangsu Section of the Huaihe River, China

**DOI:** 10.3390/ijerph17145020

**Published:** 2020-07-13

**Authors:** Kaize Zhang, Juqin Shen, Han Han, Jinglai Zhang

**Affiliations:** 1 Business School, Hohai University, Nanjing 211100, China; kzzhang@hhu.edu.cn; 2 Department of Ecosystem Science and Management, The Pennsylvania State University, State College, PA 16802, USA; 3 College of Agricultural Engineering, Hohai University, Nanjing 210098, China; jqshen@hhu.edu.cn; 4 Department of Chemistry, College of Chemistry and Chemical Engineering, Henan University, Kaifeng 475001, China; zhangjinglai@henu.edu.cn

**Keywords:** flood drainage rights, entropy weight TOPSIS model, harmonious, sustainable development

## Abstract

During the flood season, various regions in a watershed often have flood drainage conflicts, when the regions compete for flood drainage rights (FDR). In order to solve this problem, it is very necessary to study the allocation of FDR among various regions in the watershed. Firstly, this paper takes fairness, efficiency and sustainable development as the allocation principles, and comprehensively considers the differences of natural factors, social development factors, economic development factors and ecological environment factors in various regions. Then, an indicator system for allocation of FDR among regions in the watershed is established. Secondly, an entropy weight Technique for Order Preference by Similarity to Ideal Solution (TOPSIS) model is used to construct the FDR allocation model among regions in the watershed. Based on a harmony evaluation model, a harmony evaluation and comparison are carried out on the FDR allocation schemes under three different allocation principles. Finally, taking the Jiangsu section of the Huaihe River watershed as an example, the FDR of eight cities in the watershed are allocated and evaluated to see if the allocation scheme is harmonious. The results show that the allocation scheme of FDR based on the principles of fairness, efficiency and sustainable development has the highest degree of harmony, which can meet the FDR demands in various regions in the watershed, avoid the occurrence of flood drainage conflicts among regions, form an orderly flood drainage situation and promote the harmonious development of the watershed.

## 1. Introduction

Flood disaster is the result of a combination of natural exposure and human vulnerability to flood [1]. In the past three decades, the rapid development of social urbanization, the excessive emissions of anthropogenic greenhouse gases, climate change and extreme weather have occurred frequently. Combined, these factors have caused more and more flood disasters [2]. According to the fifth assessment report of the United Nations Intergovernmental Panel on Climate Change (IPCC), due to the impact of global warming, the water vapor content in the atmosphere will gradually increase; the possibility of extreme precipitation around the world will also increase [3]. Flood disaster has become one of the most serious social problems human beings must face [4]. The World Climate Organization points out that about 70% of disasters in the world are related to hydrometeorological events. Flood disaster has become an important risk factor that is restricting sustainable human development [5].

Due to abundant rainfall and dense river networks, China has become one of the countries with the most flood disasters in the world, especially in the eastern and southern regions of China [6]. Flood disasters in China are characterized by short cycles, strong intensity and massive economic losses. According to Chinese statistics, during the 2018 flood season, China’s average precipitation increased by 9.6% over the previous year, the highest in the past 20 years. Under the cloud of global warming, heavy precipitation events in China have increased significantly in the past 50 years. The length of time the precipitation falls is getting shorter and shorter, but the intensity of rainfall is gradually increasing. This increases the risk of urban waterlogging [7]. According to statistics from China’s civil affairs and water conservancy departments, during the 18 years from 1991 to 2008, the direct economic losses caused by floods in China reached 211.63 billion Yuan. That figure accounted for 48% of the total natural disaster-related economic losses. Because flood disasters have strong destructive powers, they not only cause serious economic losses but also pose a threat to people’s lives. Flood disaster has become an important factor in restricting China’s sustainable development and has aroused widespread concern throughout society. How to properly deal with flood disasters has become one of the key problems the Chinese government needs to solve, if they are to guarantee social stability and promote social harmony.

In order to cope with flood disasters, early human beings attempted to resist such disasters by constructing dams and other water conservancy projects. Subsequently, with the advancement of science and technology, the flood control standards of conservancy facilities gradually improved, playing an important role in protecting regions from flood disasters [8]. In recent years, the Chinese government has continuously increased investment in the construction of water conservancy facilities. In addition, flood drainage and flood storage projects such as drainage ditches, sluice gates, pumping stations, river networks and lakes have been developed and improved [9]. However, no matter how strong a dam is, it is impossible to completely resist or prevent flood disasters. It is obviously infeasible and uneconomical to rely solely on investing a large amount of money in building flood control conservancy facilities as a means to prevent floods [10]. Under the cloud of worrying climate change, both the amount and intensity of rainfall are gradually increasing. Existing flood storage measures and the detention capacity of water conservancy facilities also have certain limitations. Once heavy rain falls over a short time span, the flood drainage demand of each region in the watershed is concentrated for a certain period of time. This ultimately leads to the phenomenon in which the flood drainage demands of the watershed exceed the flood drainage capacity of the river channel, often resulting in flood disasters.

Then, scholars put forward a new method of combining engineering and non-engineering means to manage floods [11]. The ultimate aim of this new method is to continuously improve the ability to cope with floods, so as to adapt to and coexist with floods. Non-engineering methods emphasize that, while accepting and allowing floods to occur, they still try to reduce the losses caused by flood disasters. This is the core concept of sustainable flood risk management [12]. At present, research on non-engineering methods has mainly focused on flood risk forecasting, flood dispatching schemes and land space management. In these studies, non-engineering measures play an important role in reducing flood-related losses. However, during flood season, due to the limited flood drainage capacity of the river channel, each region in the watershed wants to try every means to drain flood waters into the river channel, in order to reduce the region’s own flood control pressure. These actions invariably cause flood drainage disputes. The reason for the flood discharge disputes between regions is the lack of any official quantification and allocation of flood discharge rights to each region in the watershed. Therefore, it is necessary to reasonably allocate flood drainage rights (FDR) to each region, in order to prevent competitive flood drainage actions between regions, reduce regional flood drainage conflicts, form an orderly flood drainage situation and reduce the losses caused by flood-related disasters in the whole watershed.

In China, flood risk management is localized. This means that local governments at all levels are responsible for local flood risk management issues [13]. In the watershed, each administrative region is distributed along the river channel and has a certain geographical order, that is, the upstream and downstream regions. The difference in geographical location means that the order of flood drainage is different for each region. Due to its geographical advantage, for example, the upstream region can drain its flood water into the river before the downstream region can.

Heavy rainfall occurring over a concentrated time span causes greater demand for flood drainage action in various administrative regions. During such times, considering only their own interests, some regions may plunder and use the resources of water conservancy projects without restraint. This phenomenon will effectively create a situation of competing for drainage flood resources. The resulting “overconsumption” and “crowding effect” causes negative external phenomena, which in turn will lead to an uneven balance of interests in the basin’s various regions. For example, in July 2016, the rainy season triggered floods. Due to drainage flooding from the upstream region in the Taihu Lake watershed, some villages in Yangxiang Town, Yixing City, which is located in the downstream region, were flooded. The result was a cumulative disaster for the city’s population of more than 130,000 people. This incident had an important impact on the property and personal safety of the people in Yixing City. The managers of all regions are “brokers”, whose primary concern is to look out for their own best interests. As such, the flood drainage conflicts between regions always exist, and problems such as disputes arising due to flood drainage have not been resolved. To solve the problem of conflicting FDR, it is necessary for the government to establish universally acceptable FDR and allocation rules. They should divide and reconfigure the FDR of various regions in the watershed and form an orderly flood drainage situation. Otherwise, the flood drainage benefits of all regions would suffer a great loss, and the overall benefits would be damaged.

The allocation of FDR within the watershed is one method of dealing with flood disasters by management means. The drainage rights proposed in this paper mainly refer to the rights of regional administrative bodies to drain flood waters into a series of water conservancy projects (such as rivers and reservoirs). These actions would happen under the permission and guidance of laws and regulations, thus ensuring each region’s safety and reducing the number of flood drainage conflicts between regions [14]. Creating FDR is an important means to control the flood drainage behavior of each region in the watershed, particularly when the flood drainage demand exceeds the regional flood drainage capacity. Yu et al. [15] believed that FDR have the characteristics of public welfare, sociality, specificity and priority. Zhang et al. [16] analyzed the necessity and feasibility of the allocation and trading of FDR in Jiangsu Province. Zhang et al. [17] used an input-output model to study the initial allocation of FDR. Zhang et al. [18], considering the natural conditions, social and economic development level and technical management, used a fuzzy analytic hierarchy process and Gini coefficient methods to study the allocation of FDR. The results of these studies provide a reference for the research contained in this paper. However, the abovementioned studies still have some limitations. First, existing studies of FDR mainly focus on the discussion of the concept and characteristics of FDR, but they lack the necessary research into the specific allocation methods of FDR. Second, these scholars only consider fairness and efficiency as the allocation principle when constructing the allocation model of FDR, but the studies lack any consideration of the principle of sustainable development. Third, in the existing studies of the allocation of FDR, scholars start from the regional economic and natural conditions (such as geographical location, watershed area, etc.), but they ignore the regional environmental factors. In the background of sustainable development, environmental factors have an important impact on the sustainable development of the watershed. If an allocation scheme ignores environmental factors, it may lead to an unreasonable allocation of drainage rights. Therefore, it is necessary to comprehensively consider the regional social and economic development, natural conditions and environmental factors when allocating FDR, in order to find a scientifically sound and reasonable allocation scheme.

**Figure 1:**
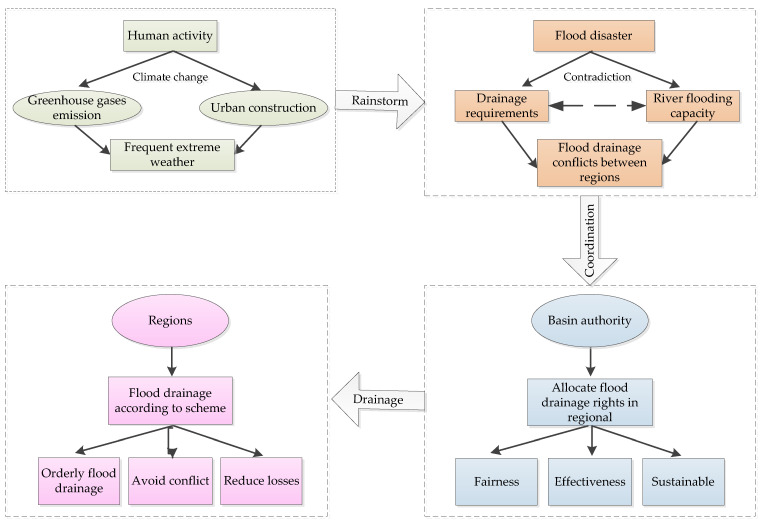
Research framework.

First, this study comprehensively considers the regional social and economic development, natural conditions and environmental conditions of each region. We then construct the indicator system of the initial allocation of FDR, including a fairness subsystem, efficiency subsystem and sustainable development subsystem. Second, an entropy weight Technique for Order Preference by Similarity to Ideal Solution (TOPSIS) model is used to build an FDR allocation model for the regions in the watershed. Then, based on a harmony evaluation model, we conduct a harmony evaluation and comparison of FDR allocation schemes under three different allocation principles. Finally, taking the Jiangsu section of the Huaihe River watershed in China as an example, the FDR are allocated and a harmonious evaluation of eight cities in the watershed is conducted. The research framework of this paper is shown in Figure 1. The study can provide a reference for solving flood drainage conflicts among regions in the watershed during flood season. This in turn can help to form an orderly flood drainage situation and avoid the occurrence of competitive flood drainage behavior among regions.

## 2. Materials and Methods

### 2.1. Study Area

**Figure 2:**
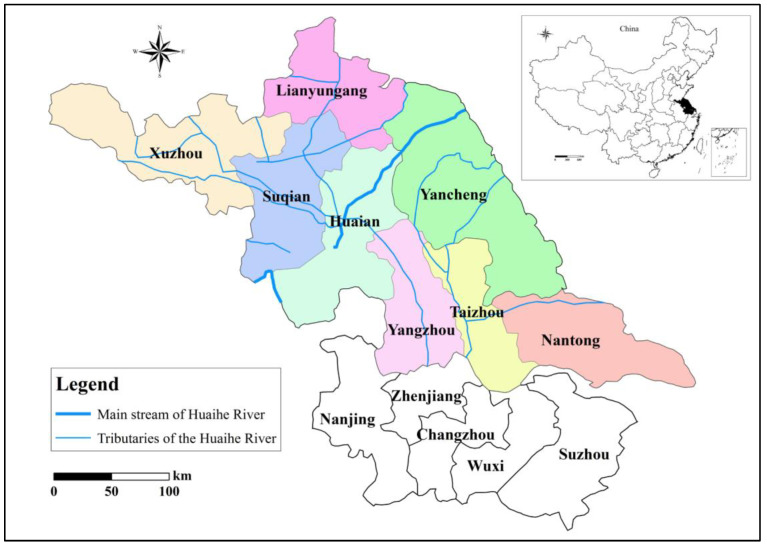
Jiangsu section of the Huaihe River watershed.

The Huaihe River is one of the seven major rivers in China, located between the Yangtze River and the Yellow River. The watershed covers an area of 270,000 km^2^ and spans five provinces in China, including Henan, Jiangsu, Hubei, Anhui and Shandong Provinces. The Huaihe River originates in Xinyang City in Henan Province and enters the Yangtze River and Yellow Sea from Jiangsu Province. There is a large drop in the elevation of the watershed, with hilly areas accounting for one-third of the watershed area; plains account for the remaining two-thirds. The Huaihe River flows through the territory, with developed water systems and numerous tributaries. Precipitation varies greatly from year to year, and rainfall occurs in a relatively concentrated time frame. The flood season occurs from June to September, with flood disasters occurring once every two to three years on average, The Huaihe River watershed is one of the watersheds with the most frequent and serious flood problems, due to the mountainous terrain in the upper reaches, relatively concentrated rainfall in the watershed and other influencing factors, including human activities. Disputes over drainage in the Huaihe River watershed continue. According to incomplete statistics, from 1949 to 1985, there were more than 400 disputes over drainage, water use and aquatic products between Shandong and Jiangsu Provinces. However, Jiangsu Province is one of the most economically developed provinces in China; the losses caused by floods to Jiangsu Province are also huge. The Huaihe River flows through eight cities in Jiangsu Province, including Xuzhou, Nantong, Lianyungang, Huai’an, Yancheng, Yangzhou, Taizhou and Suqian (see Figure 2).

### 2.2. Methods

**Figure 3:**
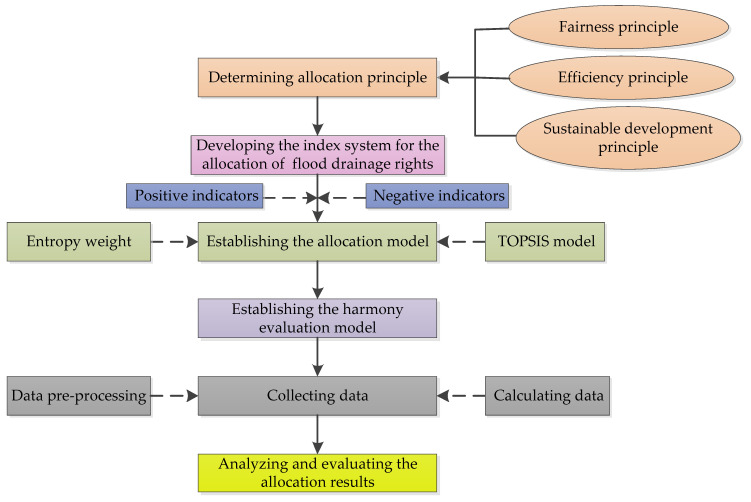
Research methods for the allocation of flood drainage rights (FDR).

This paper designs an FDR allocation scheme for the regions in the watershed (see Figure 3). The system mainly has the following allocation steps: (1) determining the principle of allocation, (2) designing the indicator system of allocating FDR, (3) establishing the allocation model of FDR between regions in the watershed, (4) establishing a harmony evaluation model, (5) collecting data and calculating the indicator data, (6) analyzing and validating the allocation result.

#### 2.2.1. Allocation Principle and Indicator System Design

Constructing a scientifically sound and reasonable indicator system is an important basis for the allocation of FDR. Different regions have different regional characteristics. It is necessary to analyze the comprehensive situation of each region in the watershed, including the natural conditions, social development, economic development, ecological environment and other factors. Starting from the three principles of fairness, efficiency and sustainable development [19], this study constructs an indicator system of initial drainage rights in the watershed.

(1) The principle of fairness

The principle of fairness refers to the fair and just treatment of each flood drainage region when allocating FDR as well as the allocation of FDR in each region according to the principle of fairness and equality [20]. Each region in the watershed is a stakeholder in the flood drainage matters and processes. At the same time, due to different natural factors, economic levels, social development levels and other factors, each region has different demands with regard to their own FDR. When designing the FDR allocation scheme, we should fully consider the natural factors, economic level, social development level and other factors of each region and design an FDR allocation scheme that is acceptable to each region. Only a fair and equal FDR allocation scheme can be successfully implemented. 

The indicator of length of flood control bank (C1) refers to the length of the embankment of the flood-retaining building in a region. The longer the C1 in a region, the longer the length of rivers flowing through the region, which means that the region is more likely to be invaded by floods. In order to treat all regions in the basin fairly, the longer the C1 is, the more FDR will be required. Population density (C2) refers to the average number of people per unit of land area over a certain period of time. The greater the C2 in a region, the greater the threat to people’s lives in the region by floods [21]. From the perspective of fairness, regions with high C2 need more FDR to ensure the safety of the people in the region. Proportion of watershed area in the region to total watershed area (C4) reflects the size of the response watershed in a region. The larger the C4 is in a certain region, the greater the harm brought by floods to that region. This means that the larger the C4, the more FDR will be required. Land area (C3) and annual rainfall (C5) both reflect the natural conditions of the region. From the perspective of fairness, the larger the C3 and C5 in the region, the more FDR is required [22].

(2) The principle of efficiency

The principle of efficiency refers to the gains (or the reduction in losses) that can be obtained by the participants in an FDR scheme when using a unit of their FDR [23]. The higher the efficiency of the FDR scheme is, the greater the benefits or the smaller the losses will be. During flood season, rivers that can accept and contain flood waters in the watershed are scarce resources. In order to bring the value of these scarce resources into full play, it is necessary to consider improving the efficiency of the use of FDR. While taking into account the principle of fairness between regions, FDR should flow to the regions with the highest use efficiency, promoting the maximization of flood drainage benefits throughout the entire watershed [24].

The indicators of built-up area (C6), per capita GDP (C7) and urbanization rate (C10) reflect the degree of economic development of a region. The larger the C6, C7 and C10 in a region, the more developed is the economy of the region. Floods with the same quantity and intensity have a greater impact on economically developed regions than economically underdeveloped regions. The income from using the FDR in economically developed regions is greater than that in economically underdeveloped areas. From the perspective of efficiency, the larger the C6, C7 and C10, the higher is the efficiency of using FDR. Density of drainage pipes (C8) reflects the strength of a region’s drainage capacity. The larger the C8, the stronger is the drainage capacity of the region, and the higher is its flood drainage efficiency [25]. Greening coverage rate of built-up areas (C9) reflects the ability of a region’s land to absorb floods.

(3) The principle of sustainable development

The principle of sustainable development refers to the development of a social economy and the simultaneous protection of the ecological environment. In other words, the development of the economy cannot be carried out at the expense of the environment. Although floods are harmful to society, they also enrich the groundwater and above-ground water resources in the watershed; they also enrich the ecological and environmental systems of the watershed [26]. The current strategic thinking in terms of flood management has shifted from preventing floods to allowing floods to occur while reducing flood disaster losses. This is the concept and essence of sustainable flood management. Thus, in the allocation of FDR, the environmental factors of the region should be comprehensively considered, in order to reduce the losses caused by flood disasters and to promote the sustainable development of society.

Sewage treatment rate (C11) refers to the capacity of a region to treat sewage. The larger the C11, the stronger the sewage treatment capacity of the region. C11 is considered to promote the protection of the ecological environment. Green area (C12) and Forest coverage (C13) reflect the condition of a region’s ecological environment. The larger the C12 and C13, the better is the environmental condition of the region. At the same time, it is less likely that floods will cause soil erosion [27]. The selection of these two indicators can promote the protection of the ecological environment in the region. Soil erosion area (C14) reflects the size of soil erosion area in a region. Soil erosion is a loss caused by rain erosion. During the flood disaster period, in areas where soil erosion is large, under the action of rainwater scouring, soil will easily flow into rivers, causing pollution to water quality [28]. From the perspective of sustainable development, this indicator should be considered.

Under the principles of fairness, efficiency and sustainable development, this paper comprehensively considers the natural conditions, social and economic development factors, ecological environment and other factors of each region. On the basis of previous studies related to FDR, this study selects 14 representative indicators that comprehensively cover the principles of fairness, efficiency and sustainable development [29]. Among them, five indicators represent fairness, five indicators represent efficiency, and four indicators represent the principle of sustainable development (see Table 1). 

#### 2.2.2. Entropy Weight Model

The indicator system of initial FDR covers 14 indicators. An entropy weight model is utilized to distinguish the degree of importance of the different indicators [30]. An entropy weight model can use the indicator data to calculate the indicator weight, which can in turn overcome the interference of human factors. As a method of subjectively determining the weights, the entropy weight model is widely used in the study of environmental analysis. The specific calculation steps of entropy weight model are as follows:

(1) Indicator data preprocessing

The allocation indicator system includes positive indicators and negative indicators. With regard to the characteristics of the indicator, a higher positive indicator indicates greater demand for drainage rights; a higher negative indicator indicates less demand for drainage rights. In order to eliminate the influence of indicator characteristics on decision results, it is necessary to standardize the selected indicators [31]. The normalization formula is:

(1) $$x_{ij}^{\prime }=\left\{ \begin{array}{l} (x_{ij}-\min\left\{x_{i}\right\})/(\max\left\{x_{i}\right\}-\min\left\{x_{i}\right\})\;x_{i}\;\mathrm{is}\;\mathrm{positive}\;\mathrm{indicator}\\ \;\\ (\max\left\{x_{i}\right\}-x_{ij})/(\max\left\{x_{i}\right\}-\min\left\{x_{i}\right\})\;x_{i}\;\mathrm{is}\;\mathrm{negative}\;\mathrm{indicator}\; \end{array}\right.$$

where $x_{ij}$ is the raw data of the indicator i in j years, $x_{ij}^{\prime }$ is the normalized value of $x_{ij}$, i=1,2,⋯,m, j=1,2,⋯,n.

(2) Calculation of the indicator weight

The formulas for determining the indicator entropy weight are as follows:

(2) $$f_{ij}=x_{ij}^{\prime }/\sum_{j=1}^{n}x_{ij}^{\prime }$$

(3) $$u_{i}=-\frac{1}{\ln n}\sum_{j=1}^{n}f_{ij}\ln f_{ij}$$

(4) $$w_{i}=(1-u_{i})/(m-\sum_{i=1}^{m}u_{i})$$

where $f_{ij}$ is the proportion of the indicator *i*, *u_i_* is the entropy value of indicator i, $w_{i}$ is the entropy weight of indicator i.

#### 2.2.3. Improved TOPSIS Allocation Model

The Technique for Order Preference by Similarity to Ideal Solution (TOPSIS) model was first established by Hwang and Yoon [32]. The TOPSIS model is one important method used to solve multi-objective decision-making problems [33]. The TOPSIS model optimizes the decision scheme by calculating the distance of each scheme from the ideal solution and the negative ideal scheme. This method can make full use of the original data of the indicator, thus ensuring that the result is more reasonable. In addition, this study improves the ideal solution of TOPSIS and introduces a virtual worst solution to better distinguish the advantages and disadvantages of indicators [34]. The specific calculation steps are as follows:

(1) Standardize the raw data.

(5) $$r_{ij}=x_{ij}^{}/\sqrt{\sum_{i=1}^{m}x_{ij}^{2}}$$

where $r_{ij}$ is the standardized value of $x_{ij}$.

(2) Construct a weighted decision matrix.

(6) $$V=(v_{ij}{)}_{mn}$$

(7) $$v_{ij}=r_{ij}\cdot w_{ij}$$

where V is the weighted decision matrix.

(3) Determine the positive and negative ideal solutions and then introduce the virtual worst solution.

(8) $$v_{i}^{+}=\left\{ \begin{array}{l} \left\{\max v_{ij}|i=1,2,\cdots ,m\right\}=\left\{v_{1}^{+},v_{2}^{+},\cdots ,v_{m}^{+}\right\}x_{i}\;\mathrm{is}\;\mathrm{positive}\;\mathrm{indicator}\\ \left\{\min v_{ij}|i=1,2,\cdots ,m\right\}=\left\{v_{1}^{-},v_{2}^{-},\cdots ,v_{m}^{-}\right\}x_{i}\;\mathrm{is}\;\mathrm{negative}\;\mathrm{indicator} \end{array}\right.$$

(9) $$v_{i}^{-}=\left\{ \begin{array}{l} \left\{\min v_{ij}|i=1,2,\cdots ,m\right\}=\left\{v_{1}^{-},v_{2}^{-},\cdots ,v_{m}^{-}\right\}x_{i}\;\mathrm{is}\;\mathrm{positive}\;\mathrm{indicator}\\ \left\{\max v_{ij}|i=1,2,\cdots ,m\right\}=\left\{v_{1}^{+},v_{2}^{+},\cdots ,v_{m}^{+}\right\}x_{i}\;\mathrm{is}\;\mathrm{negative}\;\mathrm{indicator} \end{array}\right.$$

(10) $$v_{i}^{*}=2v_{i}^{-}-v_{i}^{+}$$

where $v_{i}^{+}$ is the positive ideal solution, $v_{i}^{-}$ is the negative ideal solution, and $v_{i}^{*}$ is the virtual worst solution.

(4) Calculate the distance from the decision plan to the positive ideal and virtual worst solutions.

Distance to the positive ideal solution:

(11) $$d_{j}^{+}=\sqrt{\sum_{i=1}^{m}{\left(v_{ij}-v_{i}^{+}\right)}^{2}}$$

Distance to the virtual worst solution:

(12) $$d_{j}^{*}=\sqrt{\sum_{i=1}^{m}{\left(v_{ij}-v_{i}^{*}\right)}^{2}}$$

where $d_{j}^{+}$ is the distance from the decision plan to the positive ideal solution; $d_{j}^{*}$ is the distance from the decision plan to the virtual worst solutions.

(5) Determine the degree of closeness.

(13) $$S_{j}^{}=\frac{d_{j}^{*}}{d_{j}^{*}+d_{j}^{+}}$$

where $S_{j}^{}$ is the degree of closeness, $S_{j}^{}\in \left[0,1\right]$.

(6) Calculate the allocation ratio of FDR.

(14) $$\beta_{j}=\frac{S_{j}}{\sum_{j=1}^{n}S_{j}}$$

where $\beta_{j}$ is the allocation ratio of FDR in region j, $\sum_{j=1}^{n}\beta_{j}=1$.

#### 2.2.4. Evaluation of Harmony Degree of Allocation Scheme

The harmonious management theory refers to pursuing the harmonious coexistence of various systems in a changing environment and ultimately achieving overall sustainable development [35]. The harmonious evaluation method is an evaluation mean based on the harmonious management theory and system management theory, which is widely used to evaluate the harmony of a system [36]. The harmony of the system includes the harmony of the entire system, the mutual harmony between the subsystems within the system, and the harmony of each subsystem. Recently, the idea of harmonious management of people and water has become the new concept of water management [37]. This study uses the harmonious management theory to evaluate the rationality of the allocation scheme. The specific calculation steps of the evaluation of the degree of harmony are as follows:

(15) $$P_{k1}^{}={\left[\sum_{j=1}^{m}{\left(\beta_{j}-\max\beta_{j}\right)}^{2}\right]}^{1/2}$$

(16) $$P_{k2}^{}={\left[\sum_{j=1}^{m}{\left(\beta_{j}-\min\beta_{j}\right)}^{2}\right]}^{1/2}$$

(17) $$P_{k3}^{}={\left[\sum_{j=1}^{m}{\left(\max\beta_{j}-\min\beta_{j}\right)}^{2}\right]}^{1/2}$$

(18) $$H_{k}^{}=\frac{P_{k1}+P_{k2}}{P_{k1}+P_{k3}}$$

where $P_{k1}^{}$ is the distance between the FDR of region j and the ideal FDR in the scheme k; $P_{k2}$ is the distance between the FDR of region j and the lowest FDR in the scheme k; $P_{k3}^{}$ is the distance between the ideal FDR and the lowest FDR of region j in the scheme k. $H_{k}^{}$ is the overall harmony degree of scheme k, k=A,B,C. When k=A, the flood right allocation scheme is carried out under the principle of fairness. When k=B, the flood right allocation scheme is carried out under the principles of fairness and efficiency. When k=C, the flood right allocation scheme is carried out under the principles of fairness, efficiency and sustainability.

### 2.3. Data Source

The data of eight cities in Jiangsu section of Huaihe River watershed are all from the Jiangsu Statistical Yearbook [38], China Urban Statistical Yearbook [39], and the Jiangsu Water Conservancy Yearbook [40].

## 3. Results 

Based on the collected data of eight cities in the Jiangsu section of the Huaihe River watershed, we use an entropy weight model to calculate the weight of each indicator in the allocation of the proposed FDR indicator system. The TOPSIS model is also used to calculate the FDR of each city and to obtain the allocation scheme of FDR among the regions in the watershed. 

### 3.1. Results of Indicator Weights

According to Equations (1)–(4), the indicator entropy weights were obtained utilizing the entropy model. The entropy weight results of the indicator system for the allocation of FDR are shown in Table 2.

In the subsystem layer, the fairness subsystem has the largest weight, reaching 37.57%, followed by the environment subsystem, with its weight accounting for 31.81%. However, the weight of the environment subsystem is basically the same (only 1.19% higher) as that of the efficiency subsystem. In the indicator layer, the indicator with the largest weight (9.65%) is the proportion of watershed area in the region to total watershed area (C4). This indicator accounts for the largest proportion, because it can directly reflect the needs of the principle of fairness. In addition, the weight of population density (C2) reached 9.12%, which is also an important indicator in the fairness subsystem. The third largest weight indicator is GDP per capita (C7), at 8.62%, which is located in the principle of efficiency.

### 3.2. Result of FDR Allocation Scheme

**Figure 4:**
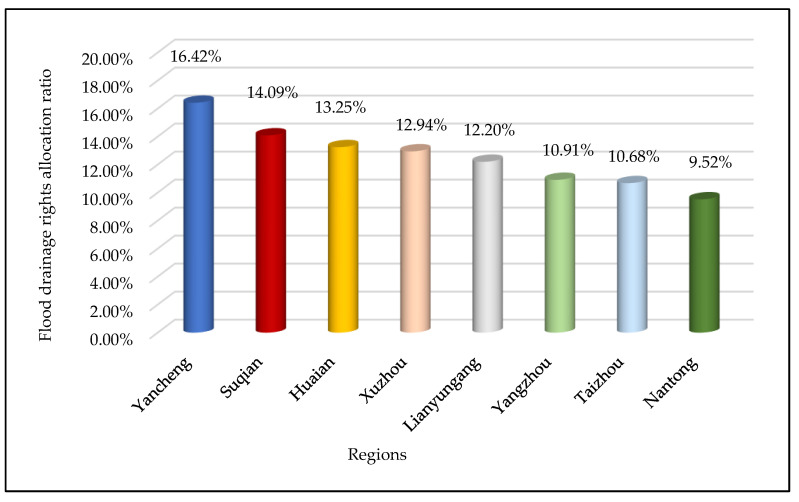
Results of FDR allocation scheme.

Based on the improved TOPSIS method, the collected data are processed through Equations (6)–(15), to obtain the following FDR allocation results, as shown in Table 3 and Figure 4.

The allocation of FDR is assigned to eight cities, from large to small, namely Yancheng, Suqian, Huai’an, Xuzhou, Lianyungang, Yangzhou, Taizhou and Nantong. The proportions of FDR assigned to each city are: 16.42%, 14.09%, 13.25%, 12.94%, 12.20%, 10.91%, 10.68% and 9.52%, respectively.

From the perspective of space, as can be seen from the geographical location of the regions in Figure 2, Nantong, Taizhou and Yangzhou are the three cities allocated the smallest proportion of FDR. All three cities are located in the central region of Jiangsu Province. The remaining five cities are located in the northern part of Jiangsu Province.

### 3.3. Result of Harmonious Evaluation

**Figure 5:**
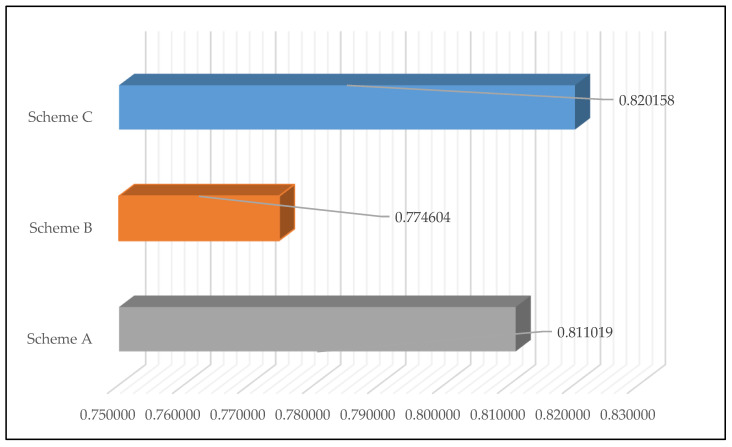
Evaluation of the harmony of the three schemes.

In order to verify the scientific quality and effectiveness of the allocation of FDR within the watershed, this paper uses Equations (15)–(18) to evaluate the results of three different allocation schemes, based on the harmonious evaluation theory. The three schemes are: Scheme A, in which the allocation of FDR are based on the principle of fairness. In Scheme B, the results of the allocation of FDR are based on the principles of fairness and efficiency. Scheme C uses the allocation results of this article. The results of the harmonious evaluation are shown in Table 4 and Table 5 and in Figure 5.

## 4. Discussion

### 4.1. Analysis of Entropy Weight

The sensitivity of each indicator value to the allocation result is determined by the weight of the indicator. The greater the weight of the indicator is, the greater the impact on the output result will be. In the subsystems, the fairness subsystem has the largest weight. This shows that the principle of fairness is the most important allocation principle in the process of FDR allocation. Fairness is also the basis and premise for the implementation of an FDR system. Only when the allocation scheme is fair and reasonable will it be accepted by all regions in the watershed. Conversely, an unfair allocation scheme will cause dissatisfaction and resistance from the parties whose interests are more likely to be damaged. Unfair schemes would also lead to flood drainage conflicts between regions. The principles of efficiency and sustainable development are the prerequisites for optimizing an allocation scheme. On the basis of the principle of fairness, improving the allocation efficiency can greatly reduce the losses caused by flood disasters. Fairness and improved efficiency mean the drainage rights will be used where they are most needed. At the same time, we also need to pay attention to environmental protection in the process of flood drainage, so as to promote sustainable development.

The indicators of the proportion of watershed area in the region to total watershed area (C4), population density (C2) and GDP per capita (C7) are considered to exert the greatest influence on the allocation of FDR. The indicator of the proportion of watershed area in the region to total watershed area (C4) accounts for the largest proportion, because this indicator can directly reflect the needs of the principle of fairness. When the watershed flows through the largest area, this region bears the greatest risks during flood season [41]. The largest areas require the largest allocation of drainage rights. In addition, when faced with a flood disaster, the watershed management department should first ensure the safety of people’s lives. On the premise of ensuring people’s safety first, efforts must be made to minimize the losses caused by flood disasters [42]. Thus, the indicator of population density is also a factor that the government needs to focus on when allocating FDR. The third largest weight indicator, GDP per capita (C7), represents the principle of efficiency. When the economic level of a region is relatively developed, the damage to the region caused by the same amount of flooding exceeds the damage to an economically underdeveloped region. Therefore, when the economically developed regions obtain more drainage rights, the use efficiency of their FDR is high.

### 4.2. Analysis of FDR Allocation Scheme

**Figure 6:**
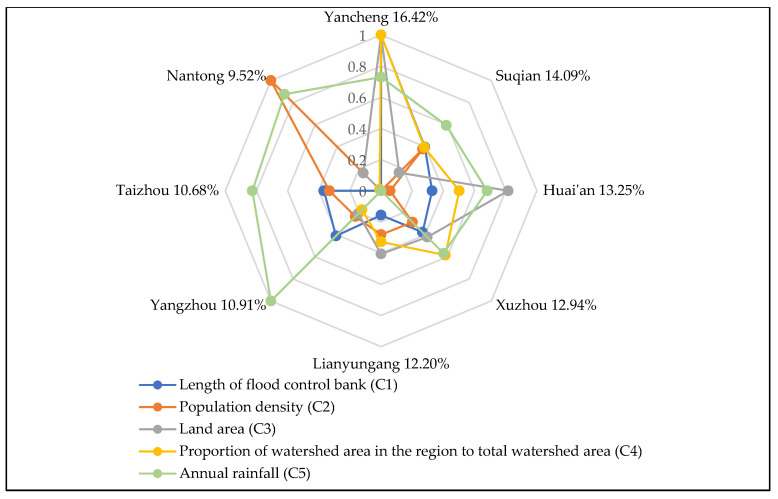
The influence of indicators of the fairness subsystem on FDR.

**Figure 7:**
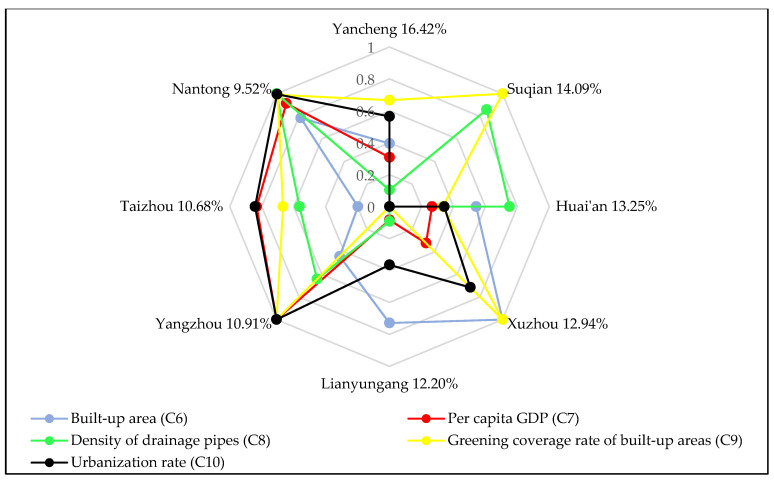
The influence of indicators of the efficiency subsystem on FDR.

**Figure 8:**
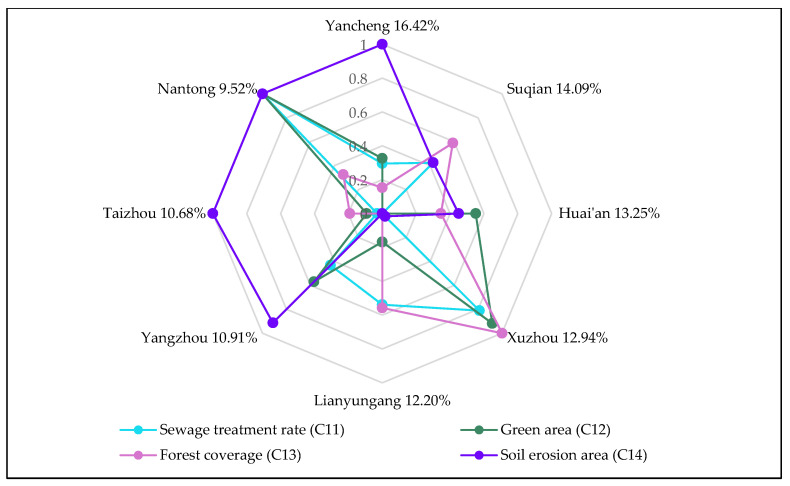
The influence of indicators of the sustainable development subsystem on FDR.

The final FDR allocation result is affected by the size of indicator value. Figure 6, Figure 7 and Figure 8 show the influence of each subsystem indicator on the regional drainage rights allocation result. The influence of each indicator on the allocation results of FDR is analyzed.

As shown in Figure 4, Yancheng City has been allocated the largest proportion (16.42%) of FDR. There are three main reasons for the allocation of Yancheng’s FDR: (1) The indicator of the proportion of watershed area in the region to total watershed area (C4) has the largest indicator value in Yancheng (as shown in Figure 6). This is the most important influencing factor in the allocation of FDR. Yancheng City has the largest proportion (25.63%) of river watershed area in the Jiangsu section of the Huaihe River watershed. In addition, Yancheng City has the longest flood bank (C1). This means that the Huaihe River watershed’s river length in Yancheng City is quite long. If flood disasters occur, Yancheng will face greater risks and come under greater flood control pressure. Starting from the principle of fairness, the larger the proportion of watershed area and the longer the flood band are, the greater the proportion of FDR allocation should be. (2) Yancheng City also has the largest land area (C3) among the eight cities, at 5131 km^2^. The larger the land area is, the greater the exposure of Yancheng to flood disasters will be. Therefore, more FDR are needed for Yancheng. (3) The indicator of soil erosion area (C14) further contributes to Yancheng’s access to a larger share of FDR. Yancheng has produced outstanding achievements in soil and water conservation. From the perspective of environmental protection, Yancheng has less probability of river water pollution caused by flood drainage after obtaining more FDR than do other cities. This will ultimately ensure the health of the rivers. To sum up, Yancheng has been allocated the largest proportion of FDR.

Suqian City, the second largest of the eight cities, obtains 14.09% of the FDR allocation. Suqian receives a large proportion of FDR for the following three reasons: (1) Suqian has a large population density (C2), the second largest among the eight cities. Due to its high population density, the greater the impact of flood disasters may be on people’s lives and safety, and the higher will be the city’s degree of vulnerability. More FDR are needed to protect people’s lives. (2) With regard to the proportion of watershed area in the region to total watershed area (C4), the watershed area of Suqian occupies a relatively large area and has large flood drainage needs. This is another reason Suqian has obtained more FDR. (3) The indicator values of density of drainage pipes (C8) and the green coverage rate of built-up areas (C9) in Suqian are relatively high. The relatively high density of drainage pipelines in Suqian indicates that Suqian has strong drainage capacity and high drainage efficiency. Suqian also has a high green coverage rate in the city’s built-up areas, indicating that the city has strong filtering, infiltration and flood water storage capacity. Therefore, Suqian has obtained more drainage rights.

Huai’an City, which is the third largest of the eight cities in terms of allocation, has received 13.25% of the FDR allocation. The following three factors have the greatest impact on the allocation of FDR in Huai’an: (1) Huai’an has a large land area (C3), the second largest of the eight cities. From the perspective of fairness, Huai’an needs more FDR to ensure the safety of the city. (2) The proportion of watershed area in Huai’an to total watershed area (C4) is also relatively large. As can be seen from Figure 2, the main stream of the Huaihe River passes through Huai’an. The watershed area of Huai’an is the third largest of the eight cities. Therefore, the flood drainage demand is also relatively large. (3) The density of drainage pipes (C8) in Huai’an City is relatively high, which means that Huai’an has strong drainage capacity and high drainage efficiency. To sum up, these are all factors that affect the allocation of FDR in Huai’an.

Xuzhou’s proportion of FDR allocation is 12.94%, which is the fourth largest allocation of the eight cities. Some factors in Xuzhou indicate a great demand for FDR. For example, Xuzhou has a relatively large watershed area, the second largest in the Jiangsu section of the Huaihe River watershed. Xuzhou also has a large built-up area (C6) and a strong density of drainage pipes (C8). The sewage treatment rate (C11) is also high. However, because Xuzhou has a low population density (C2), its economic development level is not very high; its drainage capacity is also the worst of the eight cities. The most critical reason for the poor FDR is that the soil erosion (C14) in Xuzhou is relatively serious. If Xuzhou is allocated a large proportion of FDR, this will cause greater pollution to the environment. To sum up, although some natural factors in Xuzhou indicate a large demand for FDR, there is a big gap between Xuzhou and other cities. This gap is due to a number of factors, such as Xuzhou’s lower economic development level and poor drainage efficiency and environmental protection, all of which affect the allocation result of FDR in Xuzhou.

The proportion of Lianyungang City’s FDR allocation is 12.20%, which is the fifth highest of the eight cities. All indicator data in Lianyungang are of medium level, which is why the city has obtained this mid-level allocation. The situation in Yangzhou, Taizhou and Nantong is relatively similar; they all have obtained a lesser proportion of FDR. Specifically, the proportions of FDR allocated to the three cities are 10.91%, 10.68% and 9.52%, respectively. The three cities have developed economies, high level of urban construction, good degrees of environmental protection and strong drainage capacity. However, from a geographical point of view, the three cities are located in the middle of the Huaihe River watershed and the Yangtze River watershed, all of which belongs to the central Jiangsu Province region. The proportions of watershed area in the three cities are very small (accounting for only 8.28%, 4.66% and 4.93% of the watershed area, respectively). This is because the Huaihe River flood disasters that occur in these three cities are not very serious, and the flood control pressure in the region is also small. Thus, these cities receive a lesser allocation of FDR.

### 4.3. Analysis of Harmonious Evaluation

From Table 4 and Table 5 and Figure 5, we can see that the harmony degrees of Scheme A, which is based on the principle of fairness, Scheme B, which is based on the principles of fairness and efficiency, and Scheme C, which is based on the principles of fairness, efficiency and sustainable development, are 0.81109, 0.774604 and 0.820518, respectively. This means that, compared with Scheme B and Scheme A, Scheme C shows a greater degree of harmony. According to the harmony management theory, this proves that the allocation result of Scheme C can meet the needs of the eight cities in the Jiangsu section of the Huaihe River watershed. Although Scheme A and Scheme B pursue the goals of fairness and efficiency, they ignore the factor of the ecological environment. The drainage of sewage causes frequent water pollution in river watersheds and leads to disharmony between regions, which in turn will affect the harmony of allocation schemes. As well as being allocated on the basis of fairness and efficiency, Scheme C also takes into account the influence of ecological environment factors. This makes the scheme more reasonable and scientifically sound. The proposed scheme is conducive to avoiding regional conflicts and to promoting the harmonious development of the river watershed.

## 5. Conclusions

Based on the principles of fairness, efficiency and sustainable development, this paper first constructs an indicator system for the allocation of FDR among regions in the watershed. This is done by looking for indicators that can represent the natural factors, social development factors, economic development factors and ecological environment factors of the various regions in the watershed. Then, based on the entropy weight TOPSIS method, an FDR allocation model including the various regions in the watershed is established. The harmony management theory is used to evaluate the FDR allocation result. Finally, this study takes eight regions in the Jiangsu section of the Huaihe River watershed as examples to allocate and evaluate the allocation of FDR. The research results show that the indicators of the proportion of watershed area in the region to total watershed area (C4), population density (C2), and GDP per capita (C7) are considered to exert the greatest influence on the allocation of FDR. The proportion of FDR in the eight cities in the Jiangsu section of the Huaihe River watershed is from large to small, in the order of Yancheng, Suqian, Huai’an, Xuzhou, Lianyungang, Yangzhou, Taizhou and Nantong. Based on the theory of harmonious management, the FDR allocation scheme that is based on the principles of fairness, efficiency and sustainability is considered to have the greatest degree of harmony. This finding proves that this scheme can better meet the FDR needs of various regions, as well as the management needs of watershed management agencies.

The main contributions of this paper are as follows: (1) On the basis of the principles of fairness and efficiency and considering sustainable development, an indicator system of FDR allocation is constructed. In the proposed method, environmental factors can be taken into account in the allocation of FDR. While emphasizing regional fairness and drainage efficiency, the proposed method can also promote the sustainable development of watersheds. (2) An entropy weight TOPSIS method is used to allocate the FDR among the regions in the watershed. The allocation result has passed the verification of harmony evaluation; the result is scientifically sound, reasonable and harmonious. On the premise of ensuring fairness, efficiency and sustainable development, the allocation scheme meets the needs of various regions. This study can provide new insights into FDR in watershed and flood risk management. In this paper, by allocating the FDR of various regions in the watershed, the flood drainage behavior of each region during floods can be regulated, avoiding flood drainage disputes in various regions to compete for FDR. Orderly flood drainage in each area can be achieved. This can not only reduce the losses caused by flood disasters but also promote the harmonious development between regions. In addition, this paper takes sustainable development factors into consideration when designing the FDR allocation scheme. Paying more attention to the protection of the ecological environment of the watershed when the regions drain flood is conducive to its sustainable development.

In order to ensure the proper implementation of the FDR allocation scheme, the watershed management department should use administrative means to compile river basin flood management regulations, which should clearly require all regions to drain flood, strictly following the FDR allocation scheme. At the same time, the watershed management department should monitor the amount of flood drainage in various regions in real time to prevent excessive flood drainage in those regions, giving full play to its supervision and management functions. In addition, watershed management department should formulate corresponding punishment measures to impose economic punishment and administrative punishment on illegal waterlogging areas to regulate the flood discharge behavior in each area.

The design of the indicator system is based on a comprehensive consideration of natural factors, economic and social development factors and ecological and environmental factors in Huaihe River basin. Our indicator system may not be applicable to other regions or periods. In addition, given that different regions have different natural factors, economic and social development conditions and ecological and environmental situation, other countries and regions should update the indicator system according to the characteristics of the region when studying the allocation of regional FDR, to ensure that the allocation results are more harmonious and scientific.

## Figures and Tables

**Table 1:** Indicator system for allocation of FDR.

Subsystem	Indicator	Unit	Indicator Meaning	Nature
Fairness	Length of flood control bank (C1)	km^2^	Reflects the flood control capacity of a region	+
	Population density (C2)	Persons/km^2^	Reflects the degree of population density of a region	+
	Land area (C3)	10^6^ m^2^	Reflects the size of land area in a region	+
	Proportion of watershed area in the region to total watershed area (C4)	%	Reflects the size of the response watershed in a region	+
	Annual rainfall (C5)	mm	Reflects annual rainfall in a region	+
Efficiency	Built-up area (C6)	10^6^ m^2^	Reflects the degree of urban construction in a region	+
	Per capita GDP (C7)	Yuan	Reflects the economic development degree of a region	+
	Density of drainage pipes (C8)	km/km^2^	Reflects the strength of the regional drainage capacity	+
	Greening coverage rate of built-up areas (C9)	%	Reflects the ability of a region’s land to absorb floods	+
	Urbanization rate (C10)	%	Reflects the social development of a region	+
Sustainable development	Sewage treatment rate (C11)	%	Reflects the extent of drainage pollution in a region	+
	Green area (C12)	10^4^ m^2^	Reflects the ecological environment of a region	+
	Forest coverage (C13)	%	Reflects the ecological environment of a region	+
	Soil erosion area (C14)	10^6^ m^2^	Reflects the ecological protection capacity of a region	−

**Table 2:** Results of Indicator Weights.

Subsystem	Indicator	Weight
Fairness (37.57%)	Length of flood control bank (C1)	6.54%
	Population density (C2)	9.12%
	Land area (C3)	8.57%
	Proportion of watershed area in the region to total watershed area (C4)	9.65%
	Annual rainfall (C5)	3.69%
Efficiency (30.62%)	Built-up area (C6)	5.60%
	Per capita GDP (C7)	8.62%
	Density of drainage pipes (C8)	6.93%
	Greening coverage rate of built-up areas (C9)	4.48%
	Urbanization rate (C10)	5.00%
Sustainable development (31.81%)	Sewage treatment rate (C11)	8.19%
	Green area (C12)	8.42%
	Forest coverage (C13)	7.38%
	Soil erosion area (C14)	7.82%

**Table 3:** Results of FDR Allocation Scheme.

Region	Distance from the Optimal Solution	Distance from the Worst Solution	Allocation Proportion
Yancheng	0.0018	0.0193	16.42%
Suqian	0.0042	0.0155	14.09%
Huai’an	0.0053	0.0149	13.25%
Xuzhou	0.0055	0.0143	12.94%
Lianyungang	0.0064	0.0136	12.20%
Yangzhou	0.0077	0.0120	10.91%
Taizhou	0.0080	0.0118	10.68%
Nantong	0.0094	0.0106	9.52%

**Table 4:** Results of the three allocation schemes.

Region	Scheme A	Scheme B	Scheme C	Max	Min	Max-Min
Xuzhou	12.72%	10.80%	12.94%	12.94%	10.80%	2.15%
Nantong	13.91%	13.30%	9.52%	13.91%	9.52%	4.39%
Lianyungang	13.38%	10.16%	12.20%	13.38%	10.16%	3.21%
Huai’an	12.54%	13.17%	13.25%	13.25%	12.54%	0.70%
Yancheng	11.50%	12.75%	16.42%	16.42%	11.50%	4.92%
Yangzhou	12.03%	13.89%	10.91%	13.89%	10.91%	2.98%
Taizhou	11.73%	12.74%	10.68%	12.74%	10.68%	2.07%
Suqian	12.20%	13.19%	14.09%	14.09%	12.20%	1.88%

**Table 5:** Evaluation of the harmony of the three schemes.

Index	Scheme A	Scheme B	Scheme C
P_k1_	0.05726	0.05436	0.05809
P_k2_	0.05968	0.05508	0.06085
P_k3_	0.08693	0.08693	0.08693
H_k_	0.81109	0.774604	0.820158

## References

[1] Salami R.O., Von Meding J.K., Giggins H. (2017). Urban settlements′ vulnerability to flood risks in African cities: A conceptual framework. Jàmbá J. Disaster Risk Stud..

[2] Oliver-Smith A. (2019). Climate Change, Disasters, and Development in Florida. Disasters in Paradise: Natural Hazards, Social Vulnerability, and Development Decisions.

[3] Liu Y., You M., Zhu J., Wang F., Ran R. (2019). Integrated risk assessment for agricultural drought and flood disasters based on entropy information diffusion theory in the middle and lower reaches of the Yangtze River, China. Int. J. Disaster Risk Reduct..

[4] Willems P., Arnbjerg-Nielsen K., Olsson J., Nguyen V.T.V. (2012). Climate change impact assessment on urban rainfall extremes and urban drainage: Methods and shortcomings. Atmos. Res..

[5] Associated Programme on Flood Management (2009). Integrated Flood Management: Concept Paper.

[6] Guan Y.H., Zheng F.L., Zhang P., Qin C. (2015). Spatial and temporal changes of meteorological disasters in China during 1950–2013. Nat. Hazards.

[7] Xiao M.Z., Zhang Q., Singh V.P. (2017). Spatiotemporal variations of extreme precipitation regimes during 1961–2010 and possible teleconnections with climate indices across China. Int. J. Climatol..

[8] Lebel L., Sinh B.T., Garden P., Seng S., Tuan L.A., Van Truc D. (2009). The promise of flood protection: Dikes and dams, drains and diversions. Contested Waterscapes in the Mekong Region Hydropower, Livelihoods and Governance.

[9] Gu Y. (2016). Importance of water conservancy engineering construction management and countermeasures. Agric. Technol. Inf..

[10] Liao K.H. (2012). A theory on urban resilience to floods—a basis for alternative planning practices. Ecol. Soc..

[11] Ferdous M.R., Wesselink A., Brandimarte L., Slager K., Zwarteveen M., Di Baldassarre G. (2018). Socio-hydrological spaces in the Jamuna River floodplain in Bangladesh. Hydrol. Earth Syst. Sci..

[12] De Bruijn K.M. (2004). Resilience indicators for flood risk management systems of lowland rivers. Int. J. River Basin Manag..

[13] Rubinato M., Nichols A., Peng Y., Zhang J., Lashford C., Cai Y., Lin P., Tait S. (2019). Urban and river flooding: Comparison of flood risk management approaches in the UK and China and an assessment of future knowledge needs. Water Sci. Eng..

[14] Zhang K.Z., Shen J.Q. (2019). Research on China’s Drainage Rights Trading Management under the Quasi-market—Based on the Perspective of Evolutionary Game. J. Henan Univ. (Soc. Sci.).

[15] Yu F.C., Wang Y.Z., Yuan X.J., Jiang S.M. (2014). Preliminary Study on Concept and Its Basic Characteristics of Flood Drainage Right. J. Irrig. Drain..

[16] Zhang J.S., Zhang C.S., Liu L.J., Shen J.Q., Zhang D.D., Sun F.H. (2019). Necessity and feasibility of allocation and trading of drainage rights in Jiangsu Province. Water Resour. Prot..

[17] Zhang D.D., Shen J.Q., Sun F.H., Liu B., Wang Z.Y., Zhang K.Z., Li L. (2019). Research on the Allocation of Flood Drainage Rights of the Sunan Canal Based on a Bi-level Multi-Objective Programming Model. Water.

[18] Zhang D.D., Shen J.Q., Liu P.F., Zhang Q., Sun F.H. (2020). Use of Fuzzy Analytic Hierarchy Process and Environmental Gini Coefficient for Allocation of Regional Flood Drainage Rights. Int. J. Environ. Res. Public Health.

[19] Li F., Wu F.P., Chen L.X. (2020). Harmonious allocation of carbon emission permits based on dynamic multiattribute decision-making method. J. Clean. Prod..

[20] Suresh G., Vito G.F. (2007). The tragedy of public housing: Spatial analysis of hotspots of aggravated assaults in Louisville, KY (1989–1998). Am. J. Crim. Justice.

[21] Hu P., Zhang Q., Shi P., Chen B., Fang J. (2018). Flood-induced mortality across the globe: Spatiotemporal pattern and influencing factors. Sci. Total Environ..

[22] Paprotny D., Sebastian A., Morales-Nápoles O., Jonkman S.N. (2018). Trends in flood losses in Europe over the past 150 years. Nat. Commun..

[23] Fried H.O., Shelton S.S., Lovell C.K. (1993). The Measurement of Productive Efficiency: Techniques and Applications.

[24] Karim S., Mitchell W. (2000). Path-dependent and path-breaking change: Reconfiguring business resources following acquisitions in the US medical sector, 1978–1995. Strateg. Manag. J..

[25] Zhou Q., Leng G., Su J., Ren Y. (2019). Comparison of urbanization and climate change impacts on urban flood volumes: Importance of urban planning and drainage adaptation. Sci. Total Environ..

[26] Kadir S., Badaruddin B., Nurlina N., Ridwan I., Rianawaty F. (2016). The recovery of Tabunio Watershed through enrichment planting using ecologically and economically valuable species in South Kalimantan, Indonesia. Biodiversitas.

[27] Liu W., Chen W.P., Peng C. (2014). Assessing the effectiveness of green infrastructures on urban flooding reduction: A community scale study. Ecol. Modell..

[28] Evans A.E.V., Mateo-Sagasta J., Qadir M., Boelee E., Ippolito A. (2019). Agricultural water pollution: Key knowledge gaps and research needs. Curr. Opin. Environ. Sustain..

[29] Shen J.Q., Li L., Zhang K.Z., Sun F.H. (2019). Initial Allocation of Water Discharge Right Based on Chaos Optimization-Projection Tracking. Resour. Ind..

[30] Xie T., Wang M.E., Su C., Chen W.P. (2018). Evaluation of the natural attenuation capacity of urban residential soils with ecosystem-service performance index (EPX) and entropy-weight methods. Environ. Pollut..

[31] Zhao Y.B., Wang S.J. (2015). The relationship between urbanization, economic growth and energy consumption in China: An econometric perspective analysis. Sustainability.

[32] Tzeng G.-H., Huang J.-J. (2011). Multiple Attribute Decision Making: Methods and Applications.

[33] Chen P.Y. (2019). Effects of normalization on the entropy-based TOPSIS method. Expert Syst. Appl..

[34] Sun G.D., Guan X., Yi X., Zhou Z. (2018). An innovative TOPSIS approach based on hesitant fuzzy correlation coefficient and its applications. Appl. Soft Comput..

[35] Zuo Q.T., Han C.H., Liu J., Ma J.X. (2018). A new method for water quality assessment: By harmony degree equation. Environ. Monit. Assess..

[36] Zhuang Q., Liu C.Y. (2015). The Initial Right Distribution Model of the Electric Power Industry Research based on Entropy Weight and TOPSIS Method of Carbon Emissions—Taking Jiangsu Province as an Example. Environ. Sci. Technol..

[37] Zuo Q.T., Jin R.F., Ma J.X., Cui G.T. (2015). Description and application of a mathematical method for the analysis of harmony. Sci. World J..

[38] Jiangsu Bureau of Statistics of China (2018). Jiangsu Statistics Yearbook (2018).

[39] National Bureau of Statistics of China (2018). China Urban Statistics Yearbook (2018).

[40] Jiangsu Bureau of Statistics of China (2018). Jiangsu Hydraulic Yearbook (2018).

[41] Tanaka T., Tachikawa Y., Ichikawa Y., Yorozu K. (2017). Impact assessment of upstream flooding on extreme flood frequency analysis by incorporating a flood-inundation model for flood risk assessment. J. Hydrol..

[42] Xin Y., Jiang H., Kong F., Lv L., Peng Y. (2017). Progress, Problems, and Improving Methods of Non-Engineering Countermeasures Against Urban Meteorological Disasters in China. Chin. J. Urban Environ. Stud..

